# Experiences of Stigma by Gay and Bisexual Men in Rural Oklahoma

**DOI:** 10.1089/heq.2018.0095

**Published:** 2019-05-20

**Authors:** Randolph D. Hubach, Joseph M. Currin, Zachary Giano, Hunter J. Meyers, Kyle R. DeBoy, Denna L. Wheeler, Julie M. Croff

**Affiliations:** ^1^ Center for Rural Health, Oklahoma State University-Center for Health Sciences, Tulsa, Oklahoma.; ^2^ Department of Psychology, Texas Tech University, Lubbock, Texas.; ^3^ Center for Wellness and Recovery, Oklahoma State University-Center for Health Sciences, Tulsa, Oklahoma.

**Keywords:** men who have sex with men, structural stigma, gay and bisexual men's health

## Abstract

**Purpose:** The unique experiences of men who have sex with men (MSM) residing in culturally conservative rural areas are not well represented in the scientific literature. The human immunodeficiency virus (HIV) epidemic in the United States has shifted toward rural areas where populations are dispersed and health care resources are limited.

**Methods:** We recruited 40 sexual minority men, ages 22–66, residing in rural Oklahoma for in-depth, qualitative sexual health interviews that sought to understand how cultural and social environments impacted health behaviors.

**Findings:** Participants described a stigmatizing social environment and less access to quality, sexual minority medical care within rural communities and perceived these as substantial barriers to enhancing health. Structural issues, including lack of sexual minority-affirming policies, institutional practices, and hostile cultural norms, were noted.

**Conclusions:** Results indicate the need to develop greater awareness of stigma as an etiologic factor that contributes to the health of rural sexual minority populations, specifically when it relates to provision of culturally appropriate care.

## Introduction

The unique experiences of men who have sex with men (MSM) residing in rural areas of the United States are not well represented in the scientific literature. Rural communities are typically more culturally conservative and therefore may be less welcoming to openly lesbian, gay, bisexual, and transgender (LGBT) individuals.^1–3^ Moreover, the socioeconomic conditions of rural communities mean that most individuals born into these rural communities are unable to move to areas where they would be accepted. Concurrently, the nature of the human immunodeficiency virus (HIV) epidemic in the United States has changed with a shift toward rural areas where populations are dispersed and health care resources are limited.^4^ Therefore, concurrent socioeconomic factors, geography, and cultural context are coalescing for sexual minorities living in rural communities in a way that places individuals at risk for HIV infection.^4^ Greater attention is needed to build a more comprehensive understanding of the health of marginalized populations living in rural areas of the United States, particularly among MSM.

Structural stigma, defined as societal-level conditions, cultural norms, and institutional practices that constrain opportunities, resources, and well-being for stigmatized populations, disproportionately impacts sexual minority populations and contributes to documented health disparities.^5^ MSM individuals' experiences of stigma, prejudice, and discrimination are well documented within the academic literature; however, there is a tendency to recruit samples from urban and suburban locations,^6–8^ thus ignoring the unique experiences of individuals residing in more rural areas. Expectations of rejection, concealment of identity, internalized homophobia, and discrimination constitute daily stressors that ultimately influence individual and community-level health outcomes and engagement in routine medical and mental health care.^9–13^ MSM residing in rural areas are subjected to stigma across ecological levels, however—research assessing individual-level and structural-level factors in isolation instead of evaluating synergistic relationships between social and structural factors.^3,5,12^ Such findings form the foundation for recent calls to develop interventions, grounded in empirical data, aimed at reducing individual-level and community-level stigma.^14^

Oklahoma contains two designated metropolitan areas, Oklahoma City and Tulsa; however, over half of the state's population resides in designated rural and mixed-rural areas.^15^ Research within rural Oklahoma provides opportunities to inform public health programming for other rural communities throughout the United States. Oklahoma has similar urban/rural proportions to 5 states (e.g., Oregon and Utah) and is more urban than 15 states (e.g., North Dakota and Montana). Taken together, these relatively rural states represent 20.6% of the US population.^15^

A gap in knowledge remains about MSM residing in rural areas of the United States. Minimal existing research underscores two areas of greatest need: (1) an exploration of the cultural context within rural communities, which may enhance or protect against stigma, and, following on this, (2) a more nuanced understanding of the mechanisms by which stigma, as an etiologic factor, influences health care access and utilization. To fill this gap, we conducted an exploratory qualitative study in a state with various rural municipalities to gather data that can be used to shape public health policy and program development with rural MSM.

## Methods

Qualitative data were gathered between November 2016 and May 2017. All protocols for research with human subjects were approved by the institutional review boards of the researchers' academic institutions.

### Participant recruitment and data collection

Participants were recruited through flyers placed in venues that serve MSM throughout Oklahoma (e.g., gay bars). Flyers were also placed in community venues, including faith-based organizations, medical and social service providers, public institutions (e.g., libraries), and rural-based colleges.

Participants were also recruited through electronic advertisements placed on a variety of social and sexual networking websites targeted toward MSM. Participants eligible for the interview were proficient English speakers, over the age of 18, identified as MSM, and resided in a classified rural county in Oklahoma. After consenting to the study, participants completed a one-on-one in-depth semistructured interview lasting ∼1 h and 15 min with a trained interviewer about men's sexual health. All participants giving consent completed the interview process and were compensated with a $30 retail store gift card for their participation.

### Study instruments

To gain a deeper understanding of participants' experiences, a semistructured interview guide was designed to elicit narratives from participants regarding individual-level and community-level stigma, patterns of sexual health-seeking behaviors, and determinants of sexual risk. The interview guide consisted of main questions and content-specific probes, which were based on findings from previous studies.^16–19^

### Data analyses

The interview audio was digitally recorded, transcribed, and reviewed for accuracy against the recordings. Resulting data were analyzed using a qualitative, grounded theory approach to inductively identify and interpret concepts and themes that emerged from interview transcripts.^20^ Concepts were the most basic unit of meaning from which our results developed, and related concepts were grouped into themes. This method involved multiple readings of transcripts and interview notes and analytic induction through open and axial coding of data using NVivo software (Version 11) to thematically organize transcripts. Coding was completed by two researchers independently and compared for agreement. Cohen's Kappa was calculated with all codes having k≥0.80. Open coding involved assigning conceptual codes to small sections of words, phrases, and sentences in transcripts. This was followed by axial coding, whereby we identified relationships among similar concepts and categories, which we then combined into themes. Demographic data were used to highlight occurring themes. Wherever necessary, descriptive analyses were conducted using SPSS statistical software (Version 21).

## Results

### Participant sociodemographics

Table 1 presents the demographics of the sample (*n*=40). Participants ranged in age from 22 to 66 years (mean=33.0, standard deviation=10.8) and 78% of them identified as white, not of Hispanic origin. Majority of participants identified as single (60%). Overall, 38% of the sample reported having completed an undergraduate and/or graduate degree. Notably, 58% of the total sample indicated their personal yearly income to be $30,000 or less.

**Table 1.** Sociodemographic Characteristics of the Sample (*n*=40)

	*N*
Age, years	
21–34	26
35–66	14
Education	
Less than HS	0
HS/GED	25
Bachelor's	5
Graduate	10
Ethnicity	
White	31
Biracial	3
Native American	2
Latino/Hispanic	2
Black/African American	1
Asian	1
Income	
<30,000	23
30,000–50,000	13
50,001–75,000	2
>75,000	2
Relationship	
Single	24
Partner	5
Married	9
Divorced/separated	2

Mean = 33.0, standard deviation = 10.8, range = 21–66.

HS, High School; GED, General Education Development.

### Qualitative data

Two main levels emerged in our evaluation of structural stigma among MSM in rural Oklahoma: (1) societal-level conditions and (2) cultural norms. Concurrently, there was an evident relationship between these structural factors and documented individual-level stigma. To place the themes within the context of the aforementioned identified issues, we have organized the following subsections to highlight experience with stigma across ecological levels. Quotes from data are presented to highlight and expound on connections. Pseudonyms are used to maintain participant confidentiality.

### Structural stigma: societal-level conditions

#### Policy

Participants noted that the current policy atmosphere, both national and state based, and a dearth of resources contributed to a potentially hostile environment for which MSM navigate their lives. Approximately half of the participants alluded to the lack of nondiscrimination policies for sexual orientation and gender identity, with a handful of participants acknowledging that only one city in Oklahoma and no county provides such protection within their ordinances. Instead, respondents were acutely aware that their ability to maintain employment within their community was often contingent on avoiding discussions of their sexual orientation or romantic relationships. These concerns are echoed by Xavier (28 years of age) who stated: “I'm always afraid to come out to coworkers because they might have a problem with it and then it might go to the higher-ups and you know we live in a state where it's legal to be fired for being gay.” Combined with a lack of statewide or local hate crime laws that are based on sexual orientation and religious exemption laws, several participants alluded to themselves as second-class Oklahomans. This was due to, in part, not feeling represented by their elected leaders; a form of internalized exclusion. For example, one participant posited that the lack of affirmative policy actually contributes to their invisibility: “If we're left out of laws, or demonized in them, we can't expect people at home would think differently” (Trever, 27 years of age).

#### Access to health services

A significant barrier for MSM within rural areas is access to medical and social services. Although this barrier exists for all rural community members, for MSM, it is often amplified by the lack of culturally responsive providers that account for the needs of marginalized and stigmatized populations. Specifically, rural providers interacting with these men often had religious symbolism within their patient rooms or provided patients with religious materials. Participants observed that the intersection of medical care and faith within a clinic setting more often than not led to experiences where their sexual orientation was not valued and instead was perceived as problematic. Trever (27 years of age) recalled his experience of seeing a provider while he was an emergent adult: “You walked in and there was a picture of Jesus on the wall. And when my mom told the doctor that I was bisexual, he basically told me that it was a phase and it would pass.” Mark (53 year of age) detailed how a physician overtly brought religion into the patient room when “The doctor I had gave me a card to go down and go to church.”

In addition, participants commented it was common to purposely not disclose their sexual orientation or behavior to their local physician given the potential that their physician may negatively react in a manner that could influence their care. Of those who had previously disclosed to a provider, the provider's reaction often dictated if the participant would do it again in the future. As Kevin (39 years of age) noted, “I did have one experience, a while back, where a doctor had a negative reaction when I brought up my sexuality. Since then I have been more a private person about it…sometimes not telling the truth.”

### Structural stigma: cultural norms

All participants, as demonstrated by Michael (53 years of age), noted that cultural norms within their rural communities were influenced by dominant, conservative political ideology grounded in Christian doctrine: “This is Oklahoma. This is a rural state with strong opposing conservative Christian values.”

The dominant conservative culture was viewed by participants as implicitly endorsing verbal and other forms of violence against gay, bisexual, and other MSM. A few older participants such as Howard (45 years of age) noted stories of physical violence against MSM in the community, which prompted men similar to him to hide their sexual orientation:

Growing up in that small, conservative town—there were plenty of stories of gay kids being dragged behind people's trucks in rural areas and just horribly mistreated…Had I been in a different area, like Florida or California, it might have been different, but in Oklahoma—I knew I just couldn't come out.

Although threats of physical violence from their community were of predominant concern, others indicated they had experienced verbal violence on more than one occasion. Xavier (28 years of age), as someone who moved to rural Oklahoma from another state, described how his experiences had changed: “No one has yelled faggot at me until I moved here. And it's happened once a month since then, like just walking down the street.” Such experiences have led MSM to become more aware of their surroundings given “things might happen…there is just a kind of hypervigilance of what might happen” (Zach, 26 years of age).

The intersections of multiple stigma experiences related to sexual behavior and sexual health were seen as interacting with other forms of stigma related to social identities, such as race and sexuality. Zane (26 years of age) notes how community-level views toward sexuality were exacerbated by his sexual orientation: “You have the stigma of having sex at all; because we live in rural conservative Oklahoma…then you have the stigma attached to maintaining your sexual health—like getting tested…then you have to tack on the stigma of being a sexual minority.”

#### Faith communities

Faith communities and faith leaders are often viewed as active participants in health initiatives within rural communities; however, they were also viewed by participants as gatekeepers to these sparse resources. Notably, men described the internal conflict of both being reared within a faith community and reconciling their sexual orientation. The majority of men described, “going to church and being told that homosexuality was wrong—having sex with men is wrong” (Zach, 26 years of age). Church doctrine was viewed as having permeated into familial and social relationships within their community—causing internal and interpersonal conflicts. One respondent noted in their assessment of the cultural environment:

*We live in a very conservative state, where we are basically Bible belt hard core Christian conservative, but more conservative people. My family is that way. Being gay is a sin, you are going to hell, it's just a phase, and it's a lifestyle choice* (Elias, 21 years of age).

#### Virtual communities

For many, the use of technology allowed for the connection with other men within their state. Similar to Aaron (23 years of age), men noted such outlets were vital: “If I want any interaction with another gay man, then I can wait 3 weeks until I have a conversation with one of them or I can go online and talk to one.” Although this development of a virtual community was seen as a positive outlet, an important caveat was the emphasis of both anonymity and limited relationship building (i.e., MSM appearing online only to satisfy sexual desires). For example, when discussing his social networks within his rural community, Sid (26 years of age) stated:

There are gay people out where I live but they don't want to be found…for example, when you get on dating apps you'll see them kind of around you within like 15 or 20-mile radius, but they won't have face pictures, won't have text in their profiles…they don't want any type of relationship, they just want sex. It's very isolated out here.

## Discussion

The level of social acceptance of MSM and other sexual minorities likely reflects how policies and legal statuses differ among states. Oklahoma is representative of similar relatively rural states where their remains an abundance of policies that undermine basic protection for sexual minority populations, inclusive of MSM.^21–23^ States with less supportive legal climates may also have less social acceptance compared with states with more supportive legal environments.^22^ Previous research has concluded that contact with lesbians and gay men leads to more positive evaluations of the group and more support for prosexual minority policies^24–27^; however, it is predicated on the ability of individuals to be “out” in regard to their sexual orientation. The community roles of public health, mental health, and medical providers make them uniquely positioned to advocate for and influence community norms surrounding rural MSM.^28–30^ For example, affirmative rural providers can facilitate training on how to promote openness with practices and programs in an effort to facilitate confidential disclosure.^31^

These findings mirror existing research about rural MSM who reside in cultural environments where disparities interact in a chain reaction of negative health outcomes propelled by stigma and discrimination.^32–35^ However, many of these disparities and their outcomes have been shown to be buffered by protective factors that increase an individual's resilience.^36,37^ This includes development and maintenance of social support mechanisms and being able to identify available resources.^38,39^ Contrary to their urban peers, rural MSM are more likely to experience social isolation and feelings of loneliness, which can be exacerbated in many rural environments by systemic homophobia, pressure to adhere to heteronormative expectations, and geographical estrangement from other MSM.^22,34,35,40,41^ This is exacerbated within rural communities where there remains a lack of available community resources and venues (e.g., community centers, bars, and businesses) that cater to the broader LGBT community.^18,42^

Community-based organizations, nongovernmental agencies, and advocacy organizations have traditionally utilized their resources with urban communities to facilitate community building, stakeholder engagement, and development of policy. Given that ∼12% of same-sex couples reside in rural counties,^41^ new efforts have been implemented to convene rural LGBT community members and local stakeholders.^43,44^ The Rural Pride campaign, initially a collaboration between the United States Department of Agriculture and the National Center for Lesbian Rights, utilizes day-long events within rural communities to explore LGBT-related issues that are most pressing in that community.

There is a continued need to develop greater awareness of stigma as an etiologic factor contributing to the health and well-being of rural MSM populations, specifically when relating to provisions of culturally appropriate care. Addressing stigma situated across ecological levels in an effort to improve health remains necessary, requiring that current interventions be tailored to incorporate the unique context of rural environments. The combination of both individual-level and community-level interventions provides the greatest opportunity to achieve substantial changes in health behaviors and health outcomes.^45^ Without this, social determinants may continue to negatively influence health outcomes that remain underserved and under-resourced.^36^

### Limitations

This study and the conclusions drawn from it are not without limitations. As the interview participants were recruited only from Oklahoma, we cannot assume that our results are representative of the larger population of MSM residing in other relatively rural states within the United States. In addition, our sample isolated a specific segment of the population, thus limiting our ability to observe potential pattern similarities or differences among other populations of MSM in Oklahoma, specifically by age group, race/ethnicity, and gradations of rurality. Finally, we relied on self-reported perceptions and experiences provided by participants in response to questions raised during the interview process. Self-report can reflect potential biases inherent in the use of interviews for data collection.^46^ Despite these potential limitations, our data provided much-needed formative information on stigma experienced by MSM residing in rural Oklahoma.

It is worthy to note that our sample comprised adults over the age of 18 who identified as cisgender. Because research has shown that sexual minority youth may also be impacted by minority stress,^47^ future research should investigate the interface of rurality and adolescence among youth who identify as a sexual minority. This is also the case for gender minorities (e.g., transgender individuals and nonbinary) as interviews with individuals in this group may yield different findings.

## Conclusions

This study reveals findings that can be helpful to rural public health and medical practitioners providing programming and medical services to MSM residing within their communities. MSM in Oklahoma experience substantial interactions with a stigmatizing environment, which may be similar to other states with similar rural locales. Stigma and marginalization of MSM in relatively rural states make them susceptible to structural barriers that inhibit care and access to socially supportive networks in their geographic locale. Similar to previous findings, participants identified various forms of stigma routinely encountered, such as access and barriers to culturally competent care,^31,36,48,49^ enactment of anti-LGBT public policy,^21,50,51^ and exclusion from supportive social networks (e.g., faith communities).^3,52,53^ It is clear that such needs must be considered when designing programs for MSM in rural areas of the United States.

## Abbreviations Used

HIV: human immunodeficiency virus
HS: high school
LGBT: lesbian, gay, bisexual, and transgender
MSM: men who have sex with men
